# Phosphorus Regulates the Level of Signaling Molecules in Rice to Reduce Cadmium Toxicity

**DOI:** 10.3390/cimb44090279

**Published:** 2022-09-07

**Authors:** Qiaoyu Chen, Yanyan Hu, Lijun Yang, Benguo Zhu, Feng Luo

**Affiliations:** 1 Chongqing Landscape and Gardening Research Institute, Jiulongpo, Chongqing 401329, China; 2 Chongqing Key Laboratory of Bioresource for Energy, College of Resources and Environment, Southwest University, Beibei, Chongqing 400715, China

**Keywords:** cadmium toxicity, phosphorus, antioxidant system, signal molecules

## Abstract

Phosphorus treatment can reduce Cd accumulation and Cd toxicity in rice, but alterations in the internal regulatory network of rice during this process have rarely been reported. We have removed the effect of cadmium phosphate precipitation from the hydroponic system, treated a pair of different Cd-response rice varieties with different levels of phosphorus and cadmium and examined the changes in physiological indicators and regulatory networks. The results demonstrated that phosphorus treatment significantly reduced Cd accumulation in both types of rice, although the antioxidant systems within the two types of rice produced opposite responses. Overall, 3 mM phosphorus treatment to Cd-N decreased the expression of *OsIAA17* and *OsACO1* by 32% and 37%, respectively, while increasing the expression of *OsNR2* by 83%; these three genes regulate the synthesis of auxin, ethylene, and nitric oxide in rice. IAA and NO levels in rice shoots increased by 24% and 96%, respectively, and these changes contribute to Cd detoxification. The cadmium transporter genes *OsHMA2*, *OsIRT1*, and *OsABCC1* were significantly down-regulated in Cd-N roots after triple phosphorus treatment. These data suggest that phosphorus treatment can reduce Cd accumulation and enhance Cd resistance in rice by affecting the expression of signaling molecules.

## 1. Introduction

Cadmium-contaminated soil endangers food safety and human health; cadmium exposure can cause a variety of diseases, including kidney stones, glomerular and tubular damage, Itai-Itai disease, and prostate cancer [1,2]. Rice (Oryza sativa) is the primary crop consumed by more than half of the world’s population, posing a high risk of cadmium accumulation [3]. A previous study revealed that the cadmium content of soil and rice in several areas of southeast China surpassed the national standard [4,5].

Many studies have been carried out on lowering cadmium content in crops. Numerous studies show that silicon, selenium, and phytohormones can reduce cadmium’s negative effects on plants. Date palms exposed to Cd can benefit from exogenous proline [6]. Increased Se and melatonin levels boost tomato Cd tolerance by decreasing growth inhibition, photoinhibition, and electrolyte leakage [7]. Cd was absorbed by root cell apoplasts and reduced by silicon in wheat shoots [8]. However, many studies have shown that particular rhizospheric bacteria can improve plants’ Cd tolerance. Mitra showed that Enterobacter can significantly improve morphological and biochemical traits by reducing oxidative stress and cadmium accumulation [9]. In conclusion, exogenous substances or microorganisms can be used to reduce cadmium toxicity in plants.

Phosphorus is an essential component of crop growth and is widely employed in agricultural production [10]. Phosphorus is required for cell division, root and shoot growth, flower bud differentiation, crop maturation, and food quality [11]. Cadmium enters the food chain through the production of phosphate fertilizer and is absorbed by the body after application [12]. Previous research has shown that phosphorus concentrations affect cadmium accumulation in plants. Research discovered that adding phosphate fertilizer boosted antioxidant enzyme activity and reduced cadmium in wheat [13]. Wang discovered that internal phosphorus starvation signaling had no effect on cadmium accumulation in rice [14]. Another study shows that applying phosphorus to soil can alter the soil’s microbial structure, thereby affecting the soil’s iron and nitrogen cycles and, consequently, the uptake of cadmium by plants [15]. However, the transcriptional mechanism and regulatory network by which phosphorus responds to cadmium toxicity has not been investigated; currently, research on the regulation system of phosphorus and cadmium interaction in plants is in its infancy.

Research indicates that increasing phosphorus fertilizer application can reduce cadmium uptake by crops, and that changing phosphorus fertilizer application to reduce cadmium accumulation in crops may be a practical agronomic approach. However, whether the use of phosphorus fertilizer has the same effect on different rice genotypes, whether reducing phosphorus fertilizer use results in an increase in cadmium content in rice, and the changes in hormone levels, antioxidant system, and transcriptional regulation in rice during the phosphorus process affecting cadmium uptake in rice have not been studied appropriately. We hypothesized that phosphorus treatment could affect Cd toxicity by regulating the levels of plant hormones and signaling molecules in rice; the objective of this study was to investigate this regulatory mechanism.

## 2. Materials and Methods

### 2.1. Plant Materials and Experimental Design

To investigate the effect of phosphorus treatment on cadmium toxicity in rice, four phosphorus gradient treatments were set up to simulate low, normal, high, and excessive phosphorus application; three cadmium treatment gradients were set up to simulate normal, long-term stress, and short-term stress, respectively. To confirm the generality of the effects of phosphorus treatment, two rice varieties with different Cd responses were selected for the experiment. At the end of the experiment, the physiological parameters of the two rice varieties were examined to analyze the effect of phosphorus treatment on cadmium toxicity.

The HeChuan agriculture technical extension station provided the Cd-tolerance (Cd-T) cultivar “Changxianggu” and the Cd-normal (Cd-N) cultivar “Fengyouxiangzhan”. Rice seeds were washed in a 0.5% NaClO solution for 5 min, rinsed five times with deionized water, and then immersed in deionized water at a temperature of 38 °C. After three days, the seeds were transferred to an A4 filter paper saturated with 1/4 Hoagland solution; after another three days, the solution was changed to 1/2 Hoagland solution; and after yet another three days, the germination seedlings were transferred to the hydroponics system.

To explore the effect of phosphorus on rice seedlings’ cadmium tolerance, rice seedlings were cultivated in Hoagland solution with four levels of phosphorus (0.2 mM, 1 mM, 3 mM, and 10 mM) and three levels of Cd (0 μM, 8.896 μM, and 100 μM). In total, 8.896 μM Cd is the China National Standard for heavy metal concentration in agricultural soil (GB 15618-1995). The 8.896 μM Cd treatment lasted from the start to the end of the hydroponic experiment, a total of 25 days; the 100 μM Cd treatment began on the 20th day of hydroponics and ended on the 25th (Table S1). After the preparation of the culture solution, the culture solution was filtered and tested for the cadmium level.

The hydroponic system was housed in an enclosed area with an 80% humidity level, a temperature of 30 °C, and 14 h of illumination beginning at 6:00 and ending at 20:00. Hoagland solution had the following composition: 5 mM KNO_3_, 5 mM Ca(NO_3_)_2_·4H_2_O, 2 mM MgSO_4_·7H_2_O, 0.045 mM H_3_BO_3_, 0.01 mM MnCl_2_·4H_2_O, 0.8 mM ZnSO_4_·7H_2_O, 0.3 mM CuSO_4_·5H_2_O, 0.4 mM Na_2_MoO_4_·2H_2_O, 0.02 mM FeSO_4_·7H_2_O; 0.02 mM EDTA NaOH was used to maintain the pH at 6.0; the solution was switched every three days. After that, roots and shoots were collected and dried in a 38 °C oven for seven days; the remaining samples were stored at −86 °C in a refrigerator. All of the following experiments will be performed in three replicates, while antioxidant enzymes and q-PCR will be performed in three technical replicates and three biological replicates.

### 2.2. Measurement of Cd Content

At the conclusion of the treatment, rice samples were separated into two categories: shoots and roots. After half an hour of drying at 75 °C, the samples were transferred to a 38 °C oven for seven days. After drying, the samples were immersed for 12 h in a 50 mL conical flask containing 30% hydrochloric acid and subsequently dissolved in a hydrochloric-perchloric acid system in a high-temperature electric furnace. After reducing the volume of the sample to 10 mL, the cadmium concentration was determined using flame atomic absorption spectrometry (ZA3000, Hitachi, Ltd., Tokyo, Japan).

### 2.3. Measurement of Malondialdehyde (MDA), Hydrogen Peroxide (H_2_O_2_), Superoxide Dismutase (SOD), Peroxisome (POD), and Catalase (CAT)

The malondialdehyde (MDA) content was determined using the same procedure as Wang [16]. A 0.5 g frozen shoot sample was weighed and extracted, then homogenized with 5 mL of 0.1% TCA and centrifuged at 12,000× *g* for 20 min. The MDA content was then determined by the absorbance of 450 nm, 532 nm, and 600 nm.

The modified approach was used to determine the hydrogen peroxide concentration. Leaf samples (0.5 g fresh weight, FW) were homogenized in an ice bath with trichloroacetic acid at a concentration of 3% (*w*/*v*). In total, 1 mL of the supernatant was added to 1 mL of 100 mM potassium phosphate buffer (pH 7.0) and 2 mL of 1 mol KI after centrifugation at 12,000× *g* for 15 min. At 390 nm, the absorbance was determined. The concentration of hydrogen peroxide (H_2_O_2_) was determined using a standard curve [16].

Ni’s method was used to figure out the superoxide dismutase (SOD), peroxisome (POD), and catalase (CAT) enzyme activities. The frozen simple was ground with cold phosphate buffer (pH 7.0) and the supernatants were used to determine the SOD, POD, and CAT enzyme activities [17].

### 2.4. Bioinformatics Analysis

We sequenced the transcriptomes of Cd-N rice roots and shoots treated with 8.896 M Cd; 3 mM phosphorus-treated samples were the treatment group (T) and 1 mM phosphorus-treated samples were the control group (CK). Three replicates were used for each group. The relevant bioinformatics analysis was carried out by UW Genetics.

A cDNA library was constructed for two genotype rice RNA samples and sequenced on the BGISEQ-500 platform. We removed the adapter sequences from the raw reads before assembly and removed the low-quality reads from each data set for more reliable results. For constructing the reference sequences, we merged the high-quality clean reads of the sixteen samples together, and assembled them by Trinity package. After that, sequencing reads were remapped to the reference sequences using SOAPaligner/soap2. For each gene, the expression level was measured according to the number of uniquely mapped reads using Reads Per Kilobase exon Model per Million mapped reads (RPKM), to eliminating the effect of sequencing discrepancies and different gene lengths on the calculation of gene expression. Furthermore, we selected the longest transcript to calculate the RPKM, for the genes that have more than one alternative transcript.

### 2.5. Real-Time Quantitative PCR

Total RNA from rice in different tissues was extracted using RNA RNeasy Plant Mini Kit (Qiagen, Germany). The first strand of cDNA was synthesized using the PrimeScript RT reagent kit (Takara, Dalian, China). cDNA was used for quantitative real-time PCR (qrt-pcr) to analyze gene expression, GoTaq qPCR Master Mix (Promega, Madison, WI, USA) was used. Three biological replicates and three technical replicates are performed for each genetic test. The reference gene was *OsACTIN1* (Table S2). In this study, cDNA from the roots and shoots of Cd-N rice was extracted for the assay. The sequences of the relevant genes were obtained from NCBI and the primers and synthesis conditions for qPCR were based on the tool primer3 and synthesized commercially (BGI, Beijing, China). The samples were normalized first to a selected internal control gene and then the relative gene expression levels were determined using the 2-DDCt method.

### 2.6. Measurement of IAA and NO

Chongqing Amita Biotechnology Company performed the rice IAA and NO assays in this study. Rice IAA content was measured by liquid chromatography, while nitric oxide content was measured using a modified Griess Reagent.

NO: Since nitric oxide itself is extremely unstable and is quickly metabolized into nitrate and nitrite in cells, this kit uses nitrate reductase to reduce nitrate to nitrite, and then reacts with the modified Griess Reagent to produce a colored substance with a characteristic absorption peak at 530 nm, and the total nitric oxide content in the sample to be tested can be calculated by measuring the change in its absorbance value.

IAA: Precisely weigh 0.2 g of the sample, grind it in a mortar, add 1 mL of pre-cooled 70–80% methanol solution (pH = 3.5), extract overnight at 4 °C. Centrifuge at 4 °C × 12,000× *g* for 10 min. The residue was extracted with 0.5 mL of 70–80% methanol solution at 4 °C for 2 h, centrifuged and the supernatant removed, combining the two supernatants. Evaporate at 40 °C under reduced pressure to 1/3 of the original volume, add an equal volume of petroleum ether, let stand and stratify, then extract and decolorize 2–5 times repeatedly. Add triethylamine, adjust pH = 8.0, add PVPP, shake and incubate for 20 min, centrifuge the supernatant, adjust pH to 3.0 with hydrochloric acid, extract 3 times with ethyl acetate and combine the ester phases. Evaporated to dryness at 40 °C under reduced pressure, dissolved by vortex shaking with the addition of the mobile phase solution, filtered through a needle head filter and left to measure.

### 2.7. Statistical Analysis

Data were analyzed by two-way analysis of variance (ANOVA), and treatment means were separated by Dunnett’s multiple comparisons test using GraphPad prism 9.0, all values are means with SD, *p* value style is APA (ns: *p* > 0.05, *: *p* < 0.05, **: *p* < 0.01, ***: *p* < 0.001).

## 3. Results

### 3.1. Effect of Phosphorus Treatment on the Growth Condition of Rice

Pre-experiments showed that the Cd content in the shoots of Cd-T rice was significantly higher than that of Cd-N under Cd treatment, so a follow-up experiment was conducted with Cd-T and Cd-N (Figure S1).

The dry weights of Cd-T and Cd-N were approximately equivalent under normal Cd phosphorus treatment (Figure S2A). After being subjected to Cd stress, the dry weight of Cd-T did not change significantly, while the dry weight of Cd-N decreased when treated with 8.896 μM Cd. The 0.2 mM phosphorus treatment was able to increase the biomass of Cd-T and decrease the biomass of Cd-N; the 10 mM phosphorus treatment was able to decrease both Cd-T and Cd-N biomass. The 3 mM phosphorus treatment could increase the dry weight of Cd-T and Cd-N under Cd stress (Figure S2A).

After exposure to Cd stress, the plant height of both Cd-T and Cd-N was significantly reduced. The 0.2 mM and 10 mM phosphorus treatments significantly decreased the plant height of rice, with or without Cd stress; the 3 mM phosphorus treatment was able to increase the plant height of Cd-T but had no effect on Cd-N (Figure S2B).

The effects of 0.2 mM and 10 mM phosphorus treatments on the root length of Cd-T were irregular, whereas the root length of Cd-N was significantly inhibited by various Cd treatments. When exposed to Cd stress, the 3 mM phosphorus treatment significantly increased the root length of Cd-T but had no effect on Cd-N (Figure S2C).

The 10 mM phosphorus treatment inhibited the growth of both Cd-T and Cd-N; 0.2 mM phosphorus treatment inhibited the growth of Cd-N but had no effect on Cd-T; and 3 mM phosphorus treatment alleviated the Cd toxicity of both Cd-T and Cd-N.

### 3.2. Effect of Phosphorus on Cadmium Content in Rice

**Figure 1:**
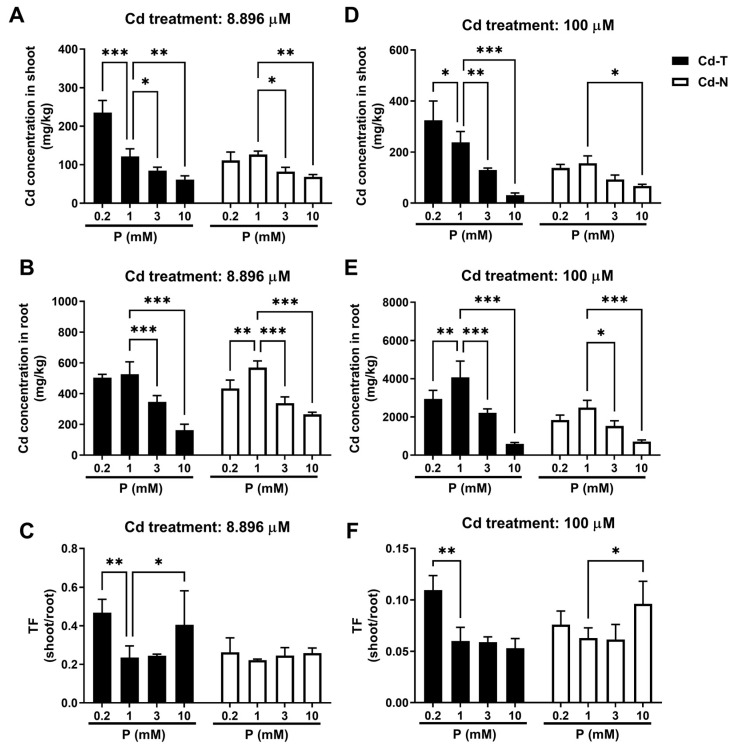
The effect of varying phosphate treatments on the Cd concentrations in rice after 8.896 μM and 100 μM Cd treatments. (**A**) Cd concentration in rice shoot under 8.896 μM cadmium treatment. (**B**) Cd concentration in rice root under 8.896 μM cadmium treatment. (**C**) TF (Transfer factor) from root to shoot under 8.896 μM cadmium treatment. (**D**) Cd concentration in rice shoot under 100 μM cadmium treatment. (**E**) Cd concentration in rice root under 100 μM cadmium treatment. (**F**) TF (transfer factor) from root to shoot under 100 μM cadmium treatment. (Values are means with SD, *n* = 3, *p* value style is APA, *p* > 0.05, *: *p* < 0.05, **: *p* < 0.01, ***: *p* < 0.001).

Under 8.896 μM long-term Cd treatment, there was no significant difference between Cd-T and Cd-N with 1 mM phosphorus treatment in Cd concentrations in the shoots and roots (Figure 1A,B). Under long-term low-Cd treatment, 3 mM and 10 mM phosphorus decreased Cd concentrations in the shoots and roots of rice; 0.2 mM phosphorus increased Cd concentrations in the shoots of Cd-T, but had no effect on Cd concentrations in the roots of Cd-T and in the shoots and roots of Cd-N (Figure 1D,E).

The Cd transport factor (shoots/roots) of two rice varieties was determined, both 0.2 mM and 10 mM phosphorus treatments significantly increased the Cd-T transport factor, but had no effect on the Cd-N transport factor (Figure 1C,F).

Under a short-term Cd treatment of 100 μM, the shoots and roots Cd contents of Cd-T were significantly higher than Cd-N; the effect of phosphorus on Cd in the tissues of both rice species was consistent under the 8.896 μM Cd treatment (Figure 1A,B,D,E). Treatment with 0.2 mM phosphorus was able to boost the transfer factor of Cd-T and Cd-N. The 0.2 mM phosphorus treatment increased both the Cd-T and Cd-N transfer factors, whereas the 10 mM phosphorus treatment only affected the Cd-N transfer factor (Figure 1C,F).

Increasing phosphorus treatment concentration decreased Cd absorption in both rice species, whereas decreasing phosphorus treatment concentration had varying effects on Cd accumulation in both rice species. Low phosphorus treatment decreased Cd in Cd-T roots, Cd-N roots, and Cd-N shoots, but increased Cd in Cd-T shoots. This was attributed to the fact that low phosphorus treatment promoted Cd translocation from Cd-T roots to Cd-T shoots.

### 3.3. Changes in the Antioxidant System

To investigate the effects of different phosphorus and cadmium treatments on the internal resistance system of rice to cadmium toxicity, antioxidant enzymes and related compounds were examined in the shoots of two rice species (Figure S3).

The MDA content represented the degree of plant cell damage, and phosphorus treatment at 10 mM caused a significantly higher MDA content in Cd-T shoots without Cd treatment, whereas phosphorus treatment at 0.2 mM and 3 mM had no significant effect (Figure S3A). The changes in antioxidant systems in plant tissues were characterized by H_2_O_2_, SOD, POD, and CAT, and phosphorus treatment had an irregular effect on the antioxidant system; however, as phosphorus treatment increased, the changes in H_2_O_2_, SOD, POD, and CAT were approximately the same in Cd-T and Cd-N (Figure S3B–E).

In general, the variation in phosphorus treatment had some effect on the antioxidant system of Cd-T and Cd-N, but the effect was not regular.

### 3.4. The Variability of the Two Rice Species

Correlation analysis and principal component analysis were used to find out how the physiological data, cadmium content, and antioxidant systems of Cd-T and Cd-N changed in phosphorus–cadmium interaction.

**Figure 2:**
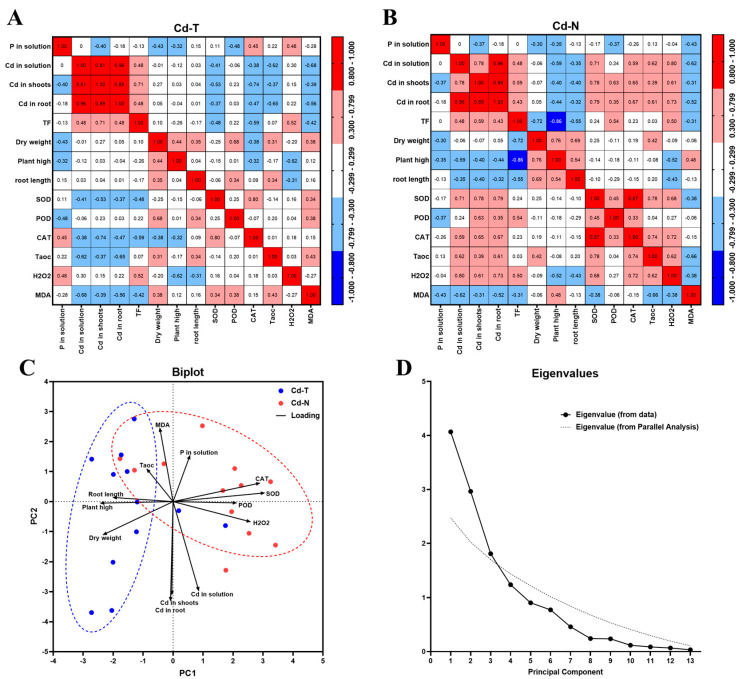
Correlation analysis and principal component analysis between Cd-T and Cd-N indicators. (**A**) Correlation analysis of Cd-T; (**B**) Correlation analysis of Cd-N; (**C**) Biplot figure of principal component analysis; (**D**) Eigenvalues.

The first column of the graph illustrates the relationship between phosphorus treatment and other indicators (Figure 2A,B). With correlation coefficients of −0.4 and −0.47, respectively, the phosphorus treatment concentrations were negatively correlated with Cd in the shoots of both rice species. Both rice species exhibited a slight negative correlation between phosphorus treatment and dry weight and plant height. Phosphorus treatment was positively correlated with SOD, CAT, and H_2_O_2_ in Cd-T, but negatively with Cd-N. Positive correlations were found between phosphorus treatment, and negative correlations with POD and MDA.

The second to sixth columns of Figure 2A,B depict the correlation between Cd and other indicators, demonstrating that the responses of Cd-T and Cd-N to Cd stress are diametrically opposed. Cd treatment had no significant effect on the dry weight, plant height, or root length of Cd-T seedlings, but had a slight negative effect on Cd-N seedlings’ plant height and root length. Cd treatment correlated negatively with the majority of Cd-T indicators, but positively with all other Cd-N indicators besides MDA.

Principal component analysis (PCA) was performed for each treatment of Cd-T and Cd-N (Figure 2C,D). Based on the eigenvalues of each PCA, PCA1 and PCA2 were selected for the analysis (Figure 2D). PCA1 was mainly related to antioxidant systems (H_2_O_2_, SOD, POD, and CAT) and growth condition (dry weight, plant height, and root length), where antioxidant systems were the opposite of physiological data. PCA2 was mainly associated with cadmium treatment, phosphorus treatment, MDA, and total antioxidant (Figure 2C). There was a significant difference in the distribution of each treatment sample of Cd-T and Cd-N in the PC1 axial direction. The samples of Cd-T were mainly distributed in the negative region of PCA1, which was in the same direction as the growth condition, while the samples of Cd-N were mainly distributed in the positive region of PCA1, which was in the same direction as the antioxidant system. The sample distributions of Cd-T and Cd-N were not significantly different in the PCA2 axis (Figure 2C).

### 3.5. Transcriptome Analysis

Rice was stressed in the 10 mM and 0.2 mM phosphorus treatments. The 8.896 μM long-term Cd treatment was designed to simulate the prevailing low contamination condition in agricultural production, while the 100 μM short-term treatment was designed to study the short-term stress response in rice. Cd-T is a specific variety with high Cd accumulation, while Cd-N is a normal variety. To investigate the intrinsic regulatory process of phosphorus treatment reducing Cd accumulation in rice, Cd-N treated with 3 mM and 1 mM phosphorus under 8.896 μM Cd treatment were selected for transcriptome analysis.

To remove the variation in phosphorus and cadmium content of the culture solution caused by cadmium phosphate precipitation, we filtered the nutrient solution and analyzed the Cd content. The results indicated that the cadmium concentration in the nutrient solution under the 1 mM and 3 mM phosphorus treatments was 0.904 mg/L and 0.895 mg/L, respectively, which was not significantly different (Figure S4).

**Figure 3:**
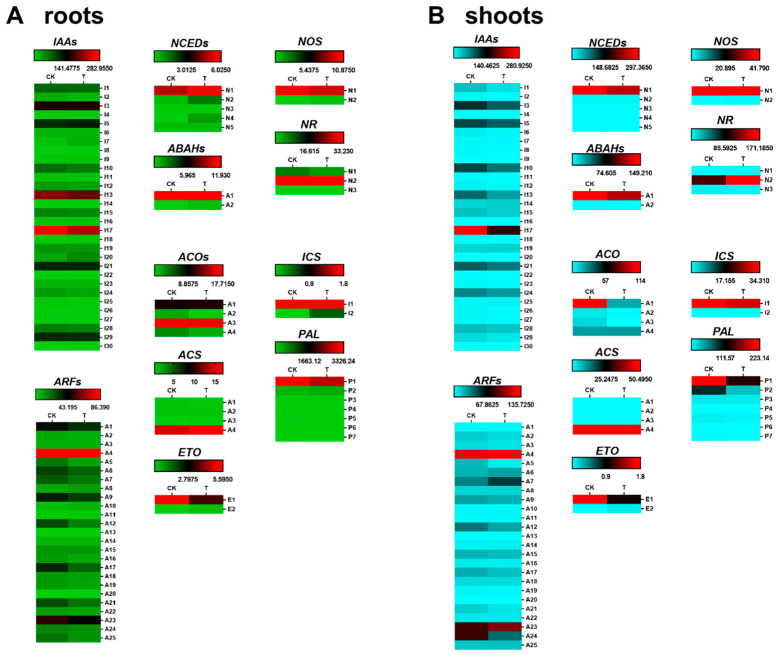
Different expression genes of signal factors (IAA, ABA, ethylene, nitric oxide, and melatonin) in root (**A**) and shoot (**B**). (CK is the control group with 1 mM phosphate and 8.896 μM cadmium, T is treatment group with 3 mM phosphate and 8.896 μM cadmium; *n* = 3).

**Figure 4:**
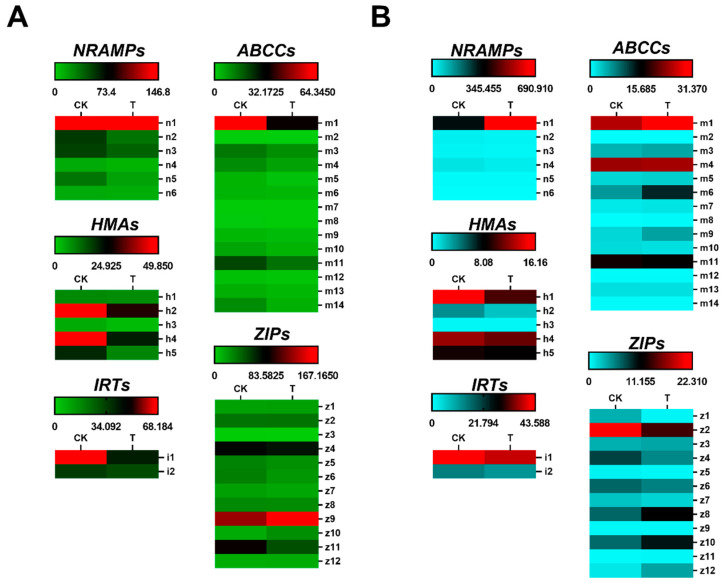
Heat map of main cadmium transporter genes in different treatments of Cd-N roots and shoots. (**A**) NRAMP, HMA, ABCC, and ZIP gene family expression in Cd-N roots. (**B**) NRAMP, HMA, ABCC, and ZIP gene family expression in Cd-N shoots. (CK is the control group with 1 mM phosphate and 8.896 μM cadmium, T is treatment group with 3 mM phosphate and 8.896 μM cadmium; *n* = 3).

To investigate the regulatory network within rice, signaling molecule and cadmium transporter genes were analyzed in the target samples (Figure 3 and Figure 4). Among the signaling molecules, we analyzed IAA, ABA, ethylene, nitric oxide, and salicylic acid-related genes. IAAs and ARFs are synergistically involved in the regulation of auxin as well as various other physiological processes; NCEDs and ABAHs are involved in the regulation of abscisic acid; ACOs, ACSs, and ETOs are involved in the regulation of ethylene; NOs and NRs are involved in the regulation of nitric oxide; and ICSs and PALs are involved in the regulation of salicylic acid. Some members of the NRAMP, HMA, IRT, ABCC, and ZIP families were identified as cadmium transport proteins or involved in the regulation of cadmium transport in *Oryza sativa* and *Arabidopsis thaliana*.

**Figure 5:**
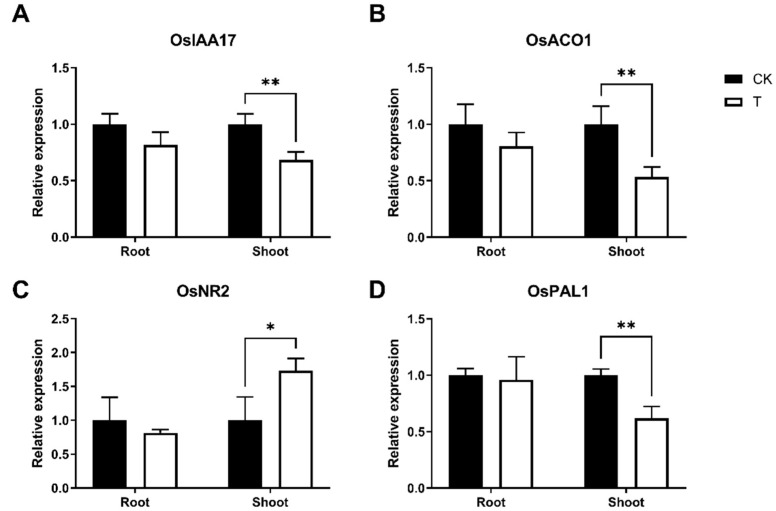
Relative expression of the signaling molecule-related genes *OsIAA17* (**A**), *OsACO1* (**B**), *OsNR2* (**C**), and *OsPAL1* (**D**) in Cd-N. (Values are means with SD, *n* = 4, *p* value style is APA, *p* > 0.05, *: *p* < 0.05, **: *p* < 0.01).

*OsIAA1, 3, 5, 10, 13, 17, 21, 29*, *OsARF1, 4, 6, 9, 12, 17, 21, 23*, *OsNCED1, 2*, *OsABAH1, OsACO1*, *3*, *OsACS4*, *OsETO1*, and *OsNOS1* were the signaling factor-related genes with the highest expression levels in Cd-N roots following 3 mM phosphorus treatment. Repressed genes included *OsIAA17*, *OsARF12, 17, 21*, *OsABAH1*, *OsACO3*, *OsETO1*, *OsNOS1*, and *OsPAL1*, and the promoted genes included *OsNCED1, 2*, *OsACS4*, and *OsICS1*. *OsIAA3, 5, 10, 13, 17, 21, 24*, *OsARF4, 7, 12, 23, 24*, *OsNCED1*, *OsACO1, 4*, *OsACS4*, *OsETO1*, *OsETO1*, *OsNOS1*, *OsNR2*, *OsICS1*, and *OsPAL1, 2* were the most highly expressed genes on Cd-N shoots. *OsIAA17*, *OsIAA17*, *OsIAA17*, *OsIAA12, 24*, *OsNCED1*, *OsABAH1*, *OsACO1*, *OsETO1*, *OsICS1*, *OsICS1*, *OsPAL1, 2* were repressed and *OsARF7, 23*, *OsNOS1*, and *OsNR2* were promoted. *OsIAA17*, *OsARF12*, *OsABAH1*, *OsACO1*, *OsETO1*, and *OsPAL1* were all repressed in both roots and shoots, while no genes were promoted. q-PCR was used to check how *OsIAA17*, *OsACO1*, *OsNR2*, and *OsPAL1* were expressed in comparison to each other (Figure 5).

**Figure 6:**
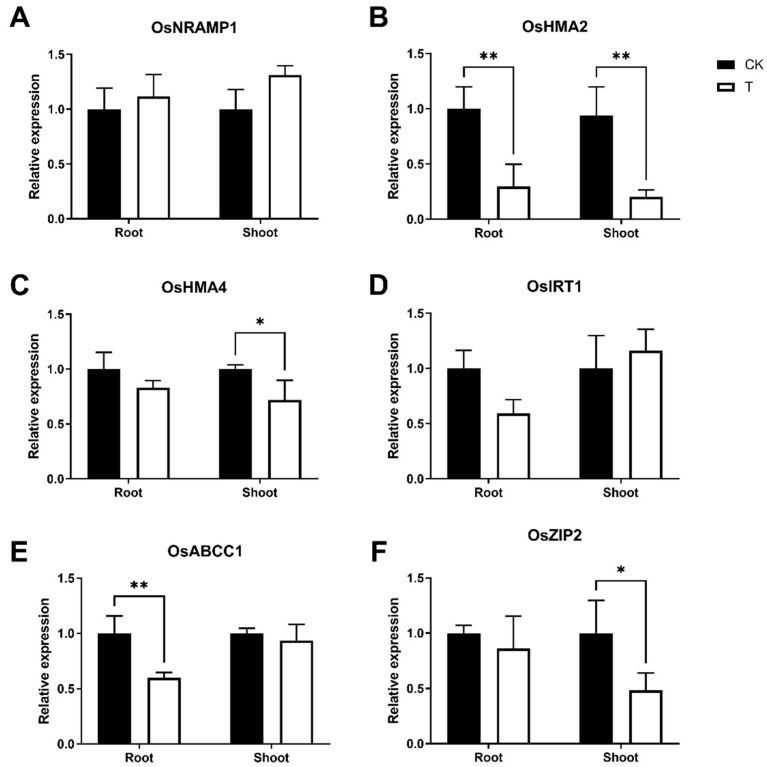
Relative expression of the transporter proteins *OsNRAMP1* (**A**), *OsHMA2* (**B**), *OsHMA4* (**C**), *OsIRT1* (**D**), *OsABCC1* (**E**), and *OsZIP2* (**F**) in Cd-N. (Values are mean with SD, *n* = 4, *p* value style is APA, *p* > 0.05, *: *p* < 0.05, **: *p* < 0.01).

Following 3 mM phosphorus treatment, *OsNRAMP1*, *OsHMA2, 4, 5*, *OsIRT1, 2*, *OsABCC1, 11*, *OsZIP4, 9, 11* were the most highly expressed genes in the roots; *OsNRAMP1*, *OsHMA2, 4, 5*, *OsIRT1*, *OsABCC1, 4, 6, 11*, *OsZIP2, 4 6, 8, 10* were the most highly expressed genes in the shoots. In roots, *OsHMA2, 4, 5*, *OsIRT1*, *OsABCC1*, *OsZIP11* were repressed, while *OsZIP9* was promoted; in shoots, *OsHMA1*, *OsIRT1*, *OsZIP2, 4* were repressed, while *OsNRAMP1*, *OsABCC1, 6*, *OsZIP8, 10* were promoted. *OsIRT1* was repressed in both roots and shoots, and no genes were promoted concurrently. We used q-PCR to confirm that the key cadmium transporter genes *OsNRAMP1*, *OsHMA2*, *OsHMA4*, *OsIRT1*, *OsABCC1*, and *OsZIP2* are regulated in their expression (Figure 6).

### 3.6. Signal Molecules Level

**Figure 7:**
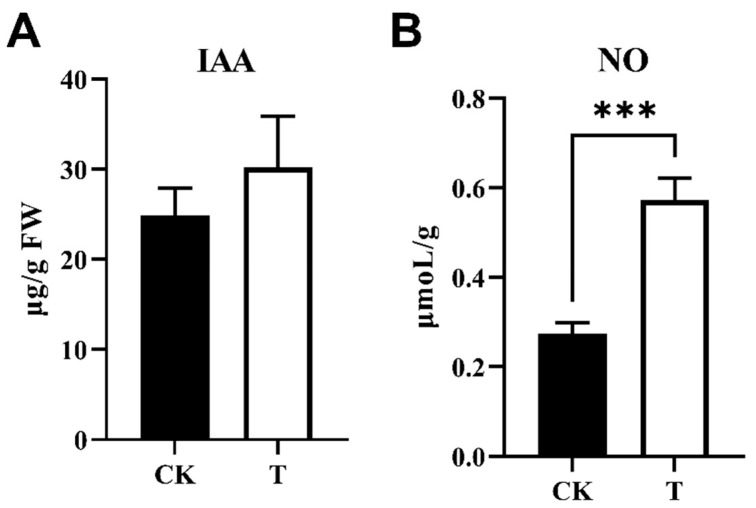
IAA (**A**) and NO (**B**) concentration in Cd-N shoots. (Values are mean with SD, IAA *n* = 5, NO *n* = 8, *p* value style is APA, *p* > 0.05, ***: *p* < 0.001).

Transcriptome and q-PCR analysis revealed that phosphorus treatment had an effect on the expression of several signaling molecule-related genes in Cd-N rice, with changes in IAA, nitric oxide, and ethylene indicating that they may act to mitigate Cd-N Cd toxicity. As a result, we measured the IAA and nitric oxide concentrations in Cd-N. The ethylene content could not be determined due to the experimental conditions. The results showed that both IAA and NO content in Cd-N shoots after 3 mM phosphorus treatment were higher than in the control; IAA content increased by 24%, which was not significant, while NO content increased by 96%, which was significant (Figure 7).

## 4. Discussion

**Figure 8:**
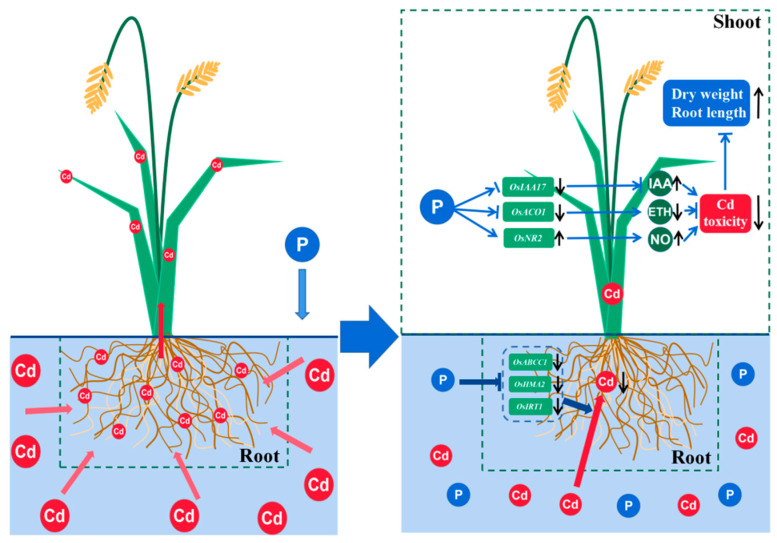
Possible mechanisms for the phosphorus reduction of cadmium toxicity in rice.

In this study, we investigated the effect of phosphorus on rice’s response to cadmium toxicity by submitting two distinct rice genotypes to a variety of phosphorus and cadmium treatments. The results indicated that increasing the phosphorus treatment can regulate the expression of signaling factors and suppress the expression of cadmium transporters in rice. We believe that higher phosphorus treatments are effective at reducing Cd accumulation in rice. Possible mechanisms for phosphorus to reduce cadmium toxicity in rice are shown in Figure 8.

Increased phosphorus treatment significantly decreased Cd accumulation in plants, as observed in wheat, spinach, and rice [18,19,20]. Previous phosphorus–cadmium experiments showed that cadmium toxicity impairs rice growth, but increasing phosphorus can repair the damage [21,22]. Cd-N responded similarly to phosphorus and cadmium treatments as other research, with Cd toxicity suppressing dry weight, plant height, and root length, but a moderate increase in phosphorus treatment could offset Cd toxicity, whereas Cd-T is a rice variety with high Cd accumulation, and Cd treatment had no significant effect on Cd-T growth (Figure 1 and Figure S2). Increased phosphorus treatment reduced the Cd content of Cd-T and Cd-N, which is consistent with previous research; however, there were significant differences in the Cd content of tissues between the two rice species when different Cd treatment patterns were used (Figure 1). Under short-term high-Cd treatment, Cd-T had a higher Cd content than Cd-N, but there was no significant difference under long-term low-Cd treatment. The difference in Cd content between Cd-T and Cd-N was comparable to the difference between the two rice varieties in Chen’s study, with Cd-T being a Cd-tolerant variety and Cd-N being a common variety [23].

Cadmium accumulation in plant tissues causes an excess of reactive oxygen, which damages plant cells. Antioxidant enzymes in plant cells scavenge the reactive oxygen species, protecting the plant from damage [24]. Rice treated with Cd will have a higher concentration of reactive oxygen such as H_2_O_2_ and O^2−^, as well as increased activity of antioxidant enzymes such as SOD, POD, and CAT [25,26]. In cadmium-toxic plants, phosphorus has an irregular effect on antioxidant enzyme activity. In Juan’s study, phosphorus treatment increased rice ASA but not GSH [25]. By contrast, Dai’s study established that increasing the phosphorus treatment significantly increased the GSH content of mangrove seedlings [21]. Arshad’s study demonstrated that phosphorus treatment can significantly increase the activity of CAT in rice [18]. Phosphorus and cadmium had no discernible effect on rice’s reactive oxygen and antioxidant enzymes (Figure S3). The response of the Cd-N antioxidant system was consistent with previous research, and the activity of the Cd-N antioxidant enzymes was positively correlated with Cd concentration and negatively correlated with phosphorus concentration (Figure 2). As Cd in Cd-T increased, antioxidant enzyme activity decreased, and phosphorus treatment increased reactive oxygen stress (Figure 2A,B).

Correlation and principal component analyses examined phosphorus’ role and the two rice species’ responses. As shown in Figure 2, increasing phosphorus treatment decreases Cd uptake in both rice species, while an excess of phosphorus inhibits rice growth; however, phosphorus’ effect on antioxidant enzymes was different between Cd-N and Cd-T. Cd had a negative correlation with Cd-N growth conditions but not with Cd-T, indicating that Cd-T is a Cd-tolerant species. In other research, Cd-N responded similarly to the wild-type control (Figure 2). Cd-T had better growth status and lower antioxidant enzyme activity, while Cd-growth N’s conditions and antioxidant system varied with phosphorus and cadmium treatment (Figure 2C). Rout’s study identified two highly Cd-tolerant rice varieties, Subhadra and Sankar, with phenotypes similar to those of Cd-T; these two rice varieties had significantly greater biomass and Cd accumulation than common varieties, but significantly higher antioxidant enzyme activities [27]. In comparison, Wang’s study discovered that, while some of the antioxidant enzyme activities of the high Cd accumulation variety S8258 decreased with increased Cd treatment similar to Cd-T, its growth was also severely inhibited by Cd toxicity [28]. The possible reason is that the majority of cadmium in Cd-T shoots is stored outside the cell, and the cell wall and membrane effectively prevent cadmium from entering the cell. According to Wu’s research, boron treatment of rice increased the resistance of rice cell walls to Cd and increased the percentage of extracellular Cd, resulting in a decrease in rice antioxidant enzyme activity [29]. However, this does not fully explain the phenotype that Cd-T antioxidant enzyme activity decreases with elevated Cd treatment. Cd-T is an intriguing variety, we are unsure whether there are any other rice varieties with similar phenotypes, and we are conducting additional research on this variety. In order to investigate the general intrinsic regulatory mechanism of phosphorus and cadmium treatment, Cd-N was used as an object of transcription regulation.

When plants were subjected to cadmium stress, IAA production by plant tissues was promoted to resist cadmium toxicity [30]. Exogenous IAA treatment of Balsam fir and Brassica napus in cadmium-contaminated soil promoted plant growth and photosynthesis and alleviated cadmium toxicity [31,32]. The function of *OsIAA17*, which is inhibited in Cd-N shoots (Figure 5A), is unknown; however, its Arabidopsis homologue, *AtIAA27*, is an auxin-inhibition gene, and the *AtIAA27* protein can bind to the auxin-tri1 complex and target it for destruction, inhibiting IAA content [33]. Phosphorus may increase IAA in shoots by suppressing *OsIAA17*, reducing cadmium toxicity. IAA in Cd-N shoots increased but did not change significantly after phosphorus treatment, indicating that IAA changes did not dominate phosphorus treatment in reducing Cd toxicity in rice (Figure 7A).

Ethylene is involved in a variety of regulatory mechanisms in plant tissues, while ACC oxidase (ACO) is involved in the final step of ethylene synthesis [34]. *OsACO1* in rice is an ethylene synthesis gene expressed in the leaf that promotes internode elongation [35]. Numerous publications have reported a significant positive correlation between *OsACO1* expression and plant ethylene levels [36]; after phosphorus treatment, *OsACO1* was greatly reduced in the shoots, showing that phosphorus may have prevented ethylene synthesis in Cd-N (Figure 5B). The application of cadmium to tomato cells caused severe oxidative stress and cell death, whereas the application of ethylene at the same time increased the cell death rate, but ethylene alone did not cause cell death [37]. Although both Cd and ethylene toxicity inhibited barley root growth, the application of an ethylene synthesis inhibitor to Cd-stressed barley roots was able to alleviate the Cd toxicity-induced growth inhibition [38]. This suggests that ethylene can increase plant Cd toxicity, whereas phosphorus treatment can inhibit the Cd-N ethylene synthesis gene, lowering Cd toxicity.

Nitric oxide is produced in plant tissues mainly through nitrate reductase (NR) and nitric oxide synthase (NOS) [39]. *OsNR2*, whose Arabidopsis homologs are *AtNIA1* and *AtNIA2*, had the highest expression of Cd-N nitric oxide-related genes in this experiment. In Arabidopsis, both *AtNIA1* and *AtNIA2* can affect nitric oxide content, and knocking out either gene resulted in a significant reduction in nitric oxide content [40]. Nitric oxide, both exogenous and endogenous, can be used to mitigate cadmium toxicity in rice by increasing the pectin and cellulose content of the rice cell wall, thereby promoting cadmium cell wall blockade and also repairing membrane damage caused by cadmium toxicity [41,42,43]. Cadmium treatment of rice and maize decreased the content of nitric oxide [44,45]. Phosphorus treatment of Cd-N increased *OsNR2* expression in shoots and NO levels, reducing Cd toxicity in rice via nitric oxide regulation (Figure 5C and Figure 7B).

In plant tissues, ICS and PAL synthesize salicylic acid. Knocking out the PAL genes in rice triples the expression of the ICS genes, but the salicylic acid content of rice tissues remains 60% lower, indicating that PAL is the primary pathway for salicylic acid synthesis in rice [46]. According to Jan’s study, salicylic acid synthesis was inhibited in rice tissues following Cd stress. Cd-N inhibited both *OsPAL1* and *OsPAL2* following phosphorus treatment (Figure 3B and Figure 5D), indicating that phosphorus treatment enhanced the inhibition of salicylic acid synthesis caused by Cd toxicity, the detoxification effect of phosphorus is not achieved through the salicylic acid pathway [47].

The genes with proven transporter or regulation of cadmium uptake in rice are *OsNRAMP1*, *OsNRAMP5*, *OsHMA2*, *OsHMA3*, *OsHMA9*, *OsABCC1*, *OsIRT1*, and *OsIRT2*, members of the ABCC and ZIP gene families, were discovered to have cadmium transport and regulatory functions in other plant species [48,49,50]. In Cd-N roots, phosphorus treatment significantly inhibited *OsHMA2*, *OsIRT1*, and *OsABCC1* expression, indicating that phosphorus treatment can help reduce Cd accumulation in rice tissues by inhibiting some Cd transporter genes when rice is stressed with Cd (Figure 6).

In this study, we showed that treating plants with phosphorus can reduce the toxicity of Cd by regulating plant signaling molecules and Cd transporter genes. However, we were unable to confirm the importance of those regulatory pathways in the detoxification process. The accumulation of Cd uptake by plants is a highly complex process that involves both rhizosphere microorganisms and root exudates, while Cd is transported in plant tissues via two distinct pathways: active transport and passive diffusion; the expression of cadmium transporter genes varies with the cadmium concentration in the plant in rice with no regulation [49,51,52,53]. Cadmium toxicity detoxification in plant tissues is more complex, involving antioxidant enzymes, phytohormones, MAPK, cell walls, vesicles, and metal chelating proteins, among others [50,54,55]. This study can only demonstrate that phosphorus treatment can affect rice signaling molecules and cadmium transporter genes to decrease the Cd toxicity and accumulation in Cd-N, which may represent only a partial pathway of phosphorus’s detoxification effect.

Increasing phosphorus application to rice may not reduce cadmium accumulation in crops. This study used three times more phosphorus to reduce cadmium in rice roots and shoots by 40%, which is impractical. Shao applied 3.86 times more phosphorus to the soil, reducing cadmium in rice by 65%. Zhao applied 3.72 times more phosphorus, reducing cadmium by 45% [20,56]. The effect was similar in other plants, with twice the amount of phosphorus applied resulting in a 40% reduction of cadmium in wheat leaves and a 45% reduction in spinach leaves [18,19]. Excessive phosphorus fertilizer application can result in severe soil hardening, as well as cadmium accumulation in the soil. Cadmium levels in soils fertilized only with nitrogen and potassium remained nearly unchanged over a 40-year period, but increased by 32% in soils fertilized with nitrogen, phosphorus, and potassium [57]. Cadmium accumulation in soils is strongly correlated with the use of phosphate fertilizers, which contain varying amounts of cadmium in the raw material for phosphate fertilizers, phosphate ore [58]. Rather than increasing phosphorus fertilizer, increasing soil phosphorus seems more realistic. Inoculating soil with arbuscular mycorrhiza fungi and organophosphorus-degrading bacteria increases available phosphorus, immobilizing heavy metals and promoting salicylic acid synthesis in plant leaves, reducing cadmium accumulation and toxicity in plants [59,60].

## 5. Conclusions

Phosphorus treatment can decrease Cd toxicity in rice via a variety of mechanisms, and phosphorus can influence the rice detoxification process by modulating signaling molecules. The phosphorus treatment process used to reduce cadmium toxicity in rice is universal and has a similar effect on rice varieties with high and low cadmium accumulation. Phosphorus treatment inhibited the expression of signaling molecules such as IAA and NO in rice tissues and increased the expression of several heavy metal transporter proteins in rice roots, lowering the cadmium content of rice and increasing its resistance to cadmium. This study provides useful information for the research of the intrinsic regulation of phosphorus–cadmium interactions in crops. Future research is needed to determine how to reduce cadmium accumulation and toxicity by increasing the available phosphorus content without increasing the total phosphorus content.

## Data Availability

The data presented in this manuscript are available on request from the corresponding author.

## Supplementary Materials

The following supporting information can be downloaded at: https://www.mdpi.com/article/10.3390/cimb44090279/s1.
