# Supplementary material for: Phosphorus Regulates the Level of Signaling Molecules in Rice to Reduce Cadmium Toxicity
Source: Curr Issues Mol Biol. 2022 Sep 7;44(9):4070–86. doi: 10.3390/cimb44090279 (PMC9497924; doi:10.3390/cimb44090279)
Supplement: Supplementary file 1 [file cimb-44-00279-s001.zip › cimb-1886269-supplementary.pdf]

Table S1. Different phosphate and cadmium treatment.

P / Cd	0.2 mM (P0)	1 mM (P1)	3 mM (P3)	10 mM (P10)
0 μM (Cd0)	P0Cd0	P1Cd0	P3Cd0	P10Cd0
8.896 μM (Cd1)	P0Cd1	P1Cd1	P3Cd1	P10Cd1
100 μM (Cd100)	P0Cd100	P1Cd100	P3Cd100	P10Cd100

Table S2. Primers for target genes and their homologs in *Arabidopsis thaliana.*

Gene name	Gene symbol	Primers (5' → 3') F	Primers (5' → 3') R	homologous gene (*Arabidopsis thaliana*)
OsIAA17	LOC4338160	GAAGATGACCAAGGACCACTACAA	GCTTCTCCGCCTTATTCACG	AtIAA27
OsACO1	LOC4347224	GAAGATGACCAAGGACCACTACAA	GCTTCTCCGCCTTATTCACG	AtACO2，AtACO3，AtACO4
OsNR2	LOC4330867	ACTGGTGCTGGTGCTTCTGGTC	CGGCTGGGTGTTGAGGGACT	AtNIA1,AtNIA2
OsPAL1	LOC4330034	TCTCGCCATCGCCAACATC	TGCCCTTGAACCCGTAGTCC	AtPAL1,AtPAL2,AtPAL3,AtPAL4
OsNRAMP1	LOC4342862	CGCTCATGCTGCTCTACG	TGTGCTTTCTCGGTGTCG	AtNRAMP1,AtNRAMP2,AtNRAMP3,AtNRAMP4,AtNRAMP5,AtNRAMP6
OsHMA2	LOC4341965	AGGACGCATCGGACAAT	GACGGCTGCCCAGATAA	AtHMA1,AtHMA2,AtHMA3,AtHMA4,AtHMA5
OsHMA4	LOC4328616	TTCTCCAAGATACCGCTACT	TGAGATCCATATTGTTCCCT	
OsIRT1	LOC4333669	GCCCTCCTTCTTCGTC	GTTTGGGTGCCGTTC	AtIRT1,AtIRT2,AtIRT3
OsABCC1	LOC4337027	GTCTACAATCCGTGCCTAC	CCACCCAATGTTTCCA	AtABCC1-15
OsZIP2	LOC4333098	AAATGGCAACGCCTCTGACCC	CGATGAGCAGCACGCTGTCG	AtZIP1-9
OsACTIN1 (reference gene)	LOC4338914	ATTCCCTCACAACAAGCG	CCATCAGGCATCTCATAGC	

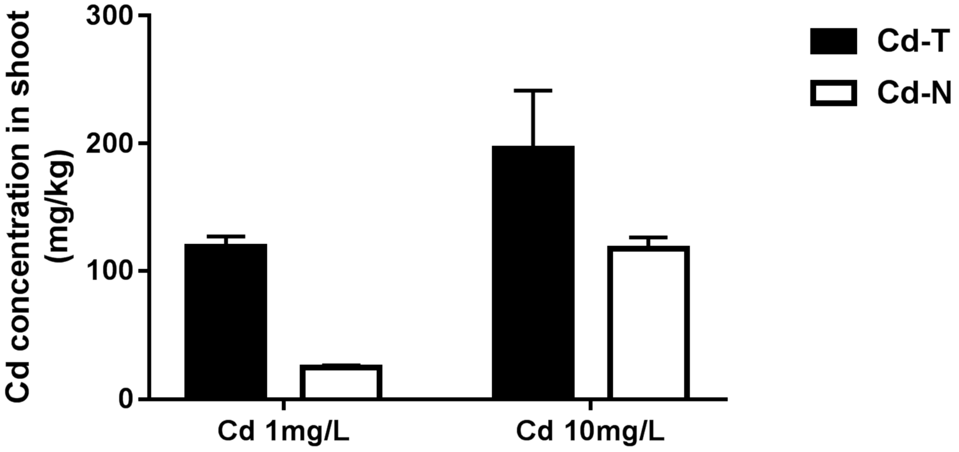

Figure S1. Cadmium content of the Cd-T and Cd-N rice grown in hydroponic system with 1mg/L and 10mg/L short-term Cd treatment. (Values are means with SD, n=3)

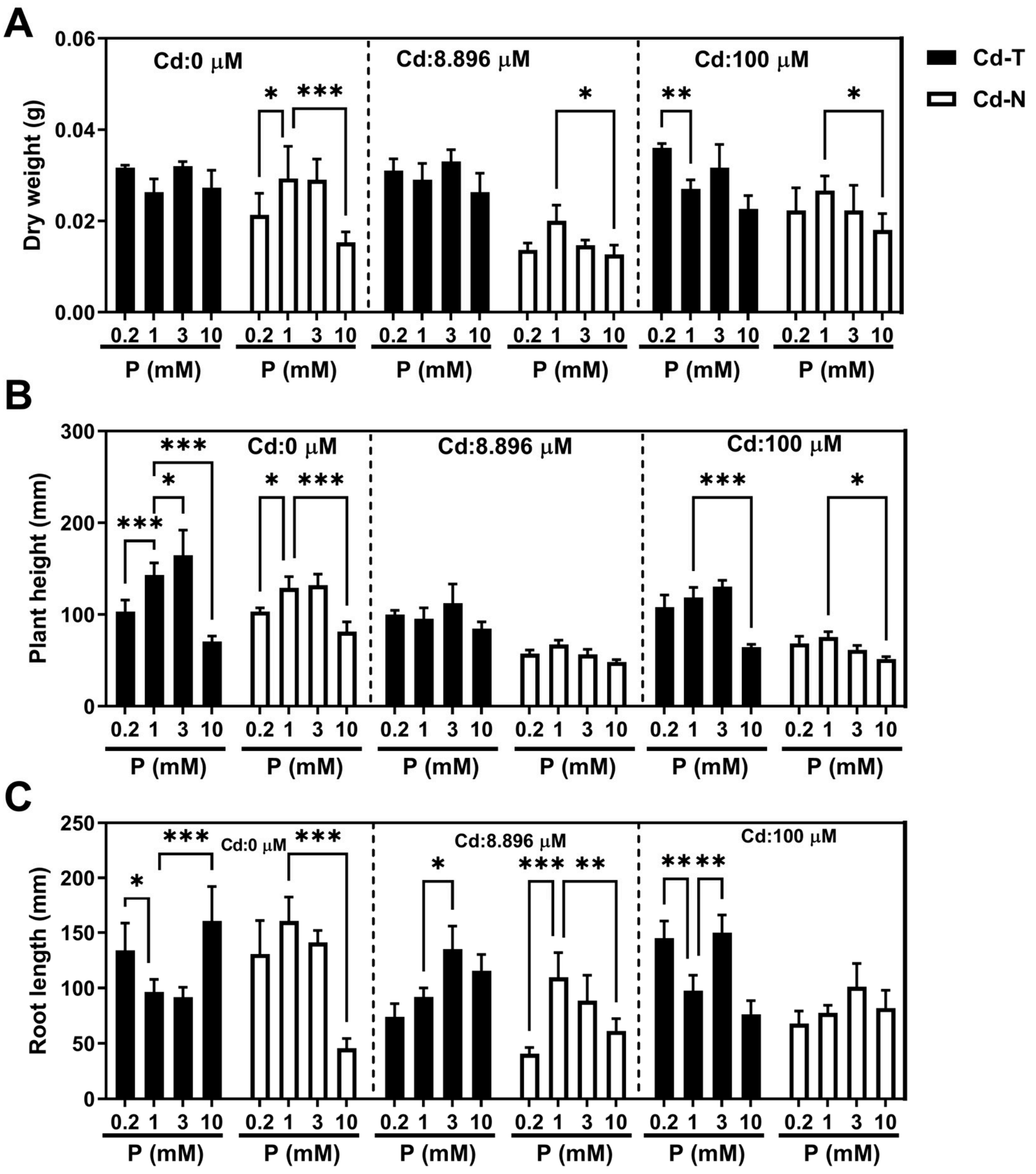

Figure S2. The effect of various phosphorus and cadmium treatments on the rice growth condition. (A) dry weight.; (B) plant height; (C) root length. (Values are means with SD, n=3, P value style is APA, ns:P>0.05, *:P<0.05, **:P<0.01, ***:P<0.001)

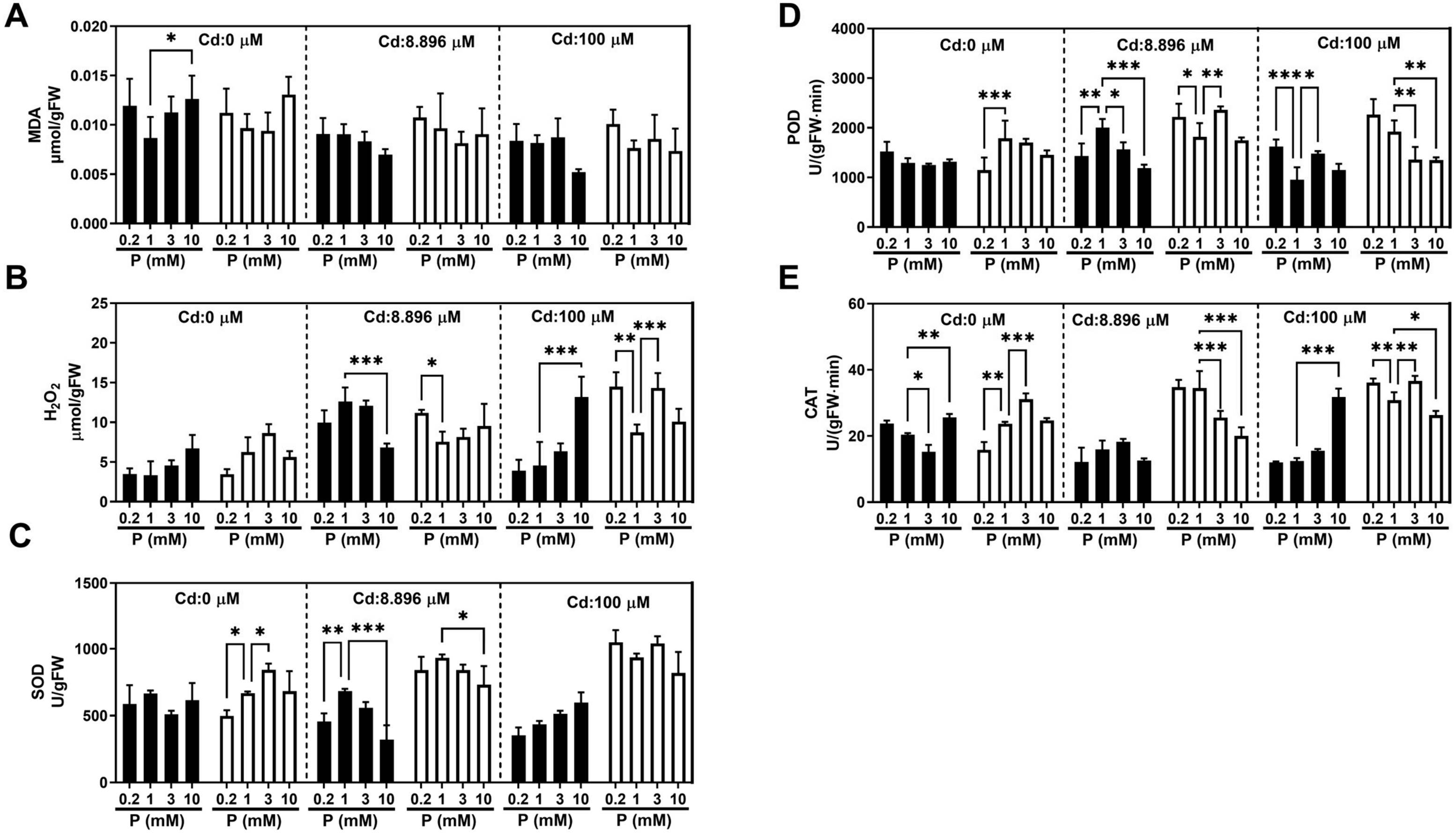

Figure S3. The effect of various phosphorus and cadmium treatments on the rice shoot peroxidation system. (A) Malonaldehyde (MDA) content; (B) hydrogen peroxide(H2O2) content; (C) Superoxide dismutase (SOD) activity; (D) Peroxidase(POD) activity; (E) Catalase(CAT) activity; (F) Total antioxidant capacity. (Values are means with SD, n=3, P value style is APA, ns:P>0.05, *:P<0.05, **:P<0.01, ***:P<0.001)

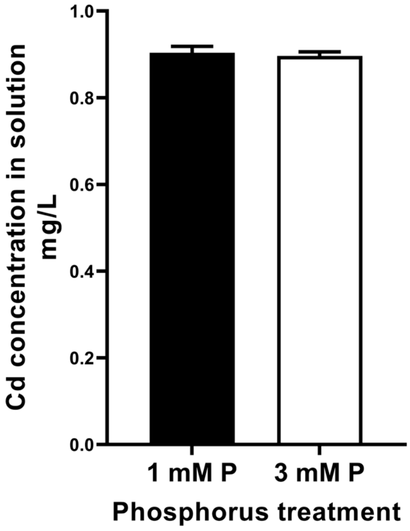

Figure S4. Cd concentration in the growing media.(Values are means with SD, n=3)
